# Mean difference in timing of first antenatal checks across regions and associated factors among pregnant women attending health facilities in Ethiopia: evidence from Ethiopian demographic health survey, 2019

**DOI:** 10.1186/s12889-023-17356-2

**Published:** 2023-12-01

**Authors:** Gemechu Chemeda Feyisa, Alemayehu Dagne, Delelegn Woyessa, Tenna Ephrem, Ausman Ahmed, Habtamu G/Senbet, Getachew Chane, Senahara Korsa Wake

**Affiliations:** 1 Department of Field Epidemiology, Faculty of Public Health, Institute of Health, Jimma University, Jimma, Ethiopia; 2 College of Natural Sciences, Mattu University, Mattu, Ethiopia; 3 Department of Biology, College of Natural Sciences, Jimma University, P. O. Box 378, Jimma, Ethiopia; 4 Oromia Health Bureau, Addis Ababa, Ethiopia; 5 College of Health Sciences, Samara University, Samara, Afar, Ethiopia; 6 College of Health Sciences, Bonga University, Bonga, Ethiopia; 7 Department of Biomedical Sciences, Jimma University, Jimma, Ethiopia; 8 College of Natural and Computational Sciences, Ambo University, Ambo, Ethiopia

**Keywords:** Antenatal checks, Mean difference, Timing, Regions, Ethiopia

## Abstract

**Introduction:**

Early initiation of antenatal care visits is an essential component of services to improve maternal health. Conducting a detailed study about the mean difference in timing at first antenatal checks across regions and factors associated with timing at first antenatal checks among women attending antenatal in Ethiopia is essential to ensure maternal and newborn health. Therefore, this study aimed to describe the mean difference in timing at first antenatal visits and associated factors among pregnant women attending different health facilities across regions in Ethiopia.

**Method:**

The Ethiopian Demographic and Health Survey (EDHS) conducted a community-based cross-sectional study in 2019. In this study, data about the timing of the first antenatal check-ups were requested from the Demographic Health Survey in February 2023, and the required variables were downloaded in SAS and SPSS formats from the data set. A total of 2935 women from nine regional states and two city administrations with an age range of 15 to 49 years were included in the study. The mean difference in timing at first antenatal check-ups, its correlation and various factors were estimated using multiple linear regressions to identify factors.

**Result:**

The majority of the 2034 (69.3%) of pregnant women who participated in the study were rural residents. The mean (± SD) age of the pregnant women was 29 (± 6.5) years. Approximately 32.5% of pregnant women visited their first antenatal check after 4 months of pregnancy. The results showed that counselling by health workers during a previous pregnancy (*p* < 0.01) significantly predicts timing at first antenatal checks in months holding previous delivery, previous antenatal care at both government and private facilities, ever attended school, and highest educational level. Timing at the first antenatal check-in months is expected to decrease by 0.99 months for every counselling session at each pregnancy. The results of the analysis suggested that the regression model significantly predicted timing at the first antenatal check (*p* = 0.001).

**Conclusion:**

The mean difference in timing at the first antenatal check in months among Ethiopian pregnant women relatively significantly varies in two regions. Previous pregnancy counselling by health workers positively influences the timing of first antenatal check-ups for subsequent antenatal check follow-ups in Ethiopia.

## Introduction

Antenatal care is a crucial component of maternal and child health and is one of the most important services connected to pregnancy that offers exceptional chances to serve pregnant women with both preventative and curative care [1]. Antenatal care may also be linked to lower maternal mother mortality because of the substantial positive correlation between the degree of care received throughout pregnancy and the utilisation of safe delivery care [2–4].

The World Health Organisation (WHO) recommends that mothers in developing countries start early prenatal care follow-up and receive at least eight antenatal care (ANC) contacts during pregnancy [4, 5]. Although the diagnosis of pregnancy-related issues unfavourable pregnancy outcomes and other difficulties require the timely commencement of ANC, most mothers initiate ANC only recently [6, 7]. As a result, maternal mortality has continued to be a global public health issue. Accordingly, one maternal death occurs every two minutes worldwide, and approximately 287,000 women and adolescent girls died from pregnancy and childbirth-related complications in 2020. The problem is even more serious in less developed countries, and approximately 70% of all maternal fatalities occur in Sub-Saharan Africa alone, with Central and Southern Asia coming in second with 17%. With 12,000 women deaths reported in Ethiopia alone by 2017, and a maternal mortality ratio (MMR) of 401 per 100,000 live births, the maternal mortality rate is still too high [8, 9], while most women began their first ANC in the third or fourth trimester of their pregnancy, maternal morbidity and death were high in underdeveloped countries, especially in Sub-Saharan Africa [10, 11]. This could be partly due to delayed first ANC visits, as several studies confirmed that the majority of pregnant women started their first ANC visits late, after 16 weeks of gestation [12–17].

Finding risk factors for the delay between the initial visit for prenatal care and delivery among pregnant women in Ethiopia has been the subject of several studies [12–19]. Investigations were conducted independently at several localities (districts) without taking into consideration the variations between the country’s regions and the random effects in the model to account for association and unobserved regional heterogeneity. When data originate from several groups or locations, heterogeneity between those regions should be taken into account. Many issues, including generalizability to the wide nation level, biased estimates of regression coefficients, and having the regression parameters estimate trend to zero, may arise if heterogeneity is ignored. To overcome these restrictions and further quantify the substantial influence of predictive factors in Ethiopia, this study evaluated the mean time of women’s first prenatal checks across areas in Ethiopia. Additionally, there was no representation at the national level and the majority of the research was done locally. The health of newborns, expectant mothers, and families will thus need to be improved, as well as general societal awareness of maternal and child morbidity and mortality. By taking into account timing in addition to the quantity of ANC visits during pregnancy, it will also serve as a reference for program designers and policy makers in the effort to lower mother and infant mortality. To examine a model that is more accurate in predicting the time to the first ANC visit in Ethiopia, this study was created to discover the determining variables for the time to the first ANC visit by taking into account different areas of the nation. To characterise the mean variation in timing at initial prenatal check-ups and related variables among pregnant women visiting various health facilities throughout regions of Ethiopia, it was worth taking to characterise variations across regions.

## Methods and materials

### Study setting and design

Data from the 2019 Mini Ethiopian Demographic and Health Surveys (EDHS), which the Central Statistical Agency collected in collaboration with the Federal Ministry of Health and the Ethiopian Public Health Institute from March 2019 to June 2019 was extracted [20]. The 2019 EDHS report contains comprehensive, detailed, outcomes of the survey at the national level, for the nine regional states and two city administrations of Ethiopia. The administration structure extends from regions to zones through the district and kebele levels. The timing of the first antenatal checks among Ethiopian women was analyzed using the EDHS data. The target population group extracted from EDHS data was women aged 15–49 years in Ethiopia. The EDHS covers data on the respondents’ socio-demographics, maternal health care, health facility services, marriage, sexual activity, child feeding customs, women’s and children’s nutritional condition, and adult and pediatric mortality.

### Data extraction procedures

During the data extraction process, the first research question was designed with the team. Based on our research question, a formal letter was requested from the DHS data archivist, along with one paragraph about the proposed project. After review of the proposed project by the DHS team, a permission letter was written from the data archivist to access and download the requested data set. Finally, we downloaded the data in SPSS and SAS formats for analysis.

### Population

The study population consisted of pregnant reproductive-age Ethiopian women. Accordingly, data from 2935 pregnant mothers were extracted. Among 2935 pregnant women, 17 did not know the dates of ANC visits. All pregnant women with the age group of 15–49 years at their first ANC visit were included into the data analysis.

### Sampling methods

The EDHS 2019 sample was divided into two groups before being chosen. Twenty-one sample strata were produced after stratifying each region into urban and rural regions. A total of 305 enumeration areas (EAs) (93 in urban areas and 212 in rural regions) were chosen in the first stage, with the likelihood inversely correlated with EA size. From the newly formed household listing, a mixed number of 30 homes per cluster was chosen in the second step with an equal likelihood of systematic selection. The EDHS 2019 report contains information on the precise sampling process [20]. A total of 2935 pregnant women were enrolled in this analysis.

### Eligibility criteria

#### Inclusion

All pregnant women with the age group of 15–49 years at their first ANC visit were included after informed consent was obtained from all subjects and/or their legal guardian(s).

#### Exclusion criteria

Women with unrecorded or unknown gestational age at the time of their first ANC visit were excluded from the study, as were women with many missing variables. Moreover, those variables with many missing values were excluded.

### Study variables

#### Dependent variable

The dependent variable was the timing of the first antenatal care in months.

#### Independent variables

Different independent variables were considered in this study to determine correlations and factors associated with timing at first antenatal care, including socio-demographic factors (age, religion, educational level, place of residence, and region) and service-related factors (history of previous vaccination, antenatal care facility type, delivery by caesarean section, place of delivery, counselling during a previous pregnancy).

### Data processing and analysis

Data from the EDHS 2019 individual (women) record folder were extracted. Data were recoded, cleaned, and edited to fit the purpose of this secondary analysis. Listing and sorting the variables were performed to find any missing values.

For the descriptive statistics, quantitative variables were summarized using the mean and standard deviation. Meanwhile, categorical variables were presented as frequencies and percentages.

Multiple correlation analysis was performed. One-way analysis of variance (ANOVA) was used. Meanwhile, the Kruskal-Wallis test was used instead of ANOVA when data was skewed or violated the assumptions of the parametric test.

Multiple linear regression models were used to determine the factors associated with the timing of the first antenatal check among regions in Ethiopia. In particular, the saturated model containing all the variables being investigated was used, with variables with a *p*-value of <0.05 considered statistically significant. Assumptions of the test, such as homogeneity of variance, independence, multicollinearity, normality, and linearity were checked. Logarithmic transformation was performed for variables that violated the normality assumption. SAS 9.4 version software was used to perform the analyses.

## Results

### Socio-demographic characteristics of women attending health facilities in Ethiopia

A multilevel cross-sectional study of timing at the first antenatal checkin months among women attending antenatal check in Ethiopia showed that 2935 pregnant women were included in this study. In characterization of the age of women, 170 (5.8%) were age group 15–19 years, 614(21%) were in the age group 20–24 years, 915(32.5%) were in the age group 25–29 years, 603(20.5%) were in the age group 30–34 years, 396(13.5%) were in the age group 35–39 years, 154(5.2%) were in the age group 40–44 years and 45(1.5%) were in the age group 45–49 years. The mean (± SD) age of the pregnant women was 29 (± 6.5) years. Regarding timing at the first antenatal check, approximately 56.3% of pregnant women visited their first antenatal check after 3 months of pregnancy or 12 weeks of gestational age (GA).

The majority of pregnant women, 69.3% (2034/295) were rural residents. Approximately, 38.4% (9782/2034) of rural pregnant women attended their first antenatal check after 4 months of their gestational age, whereas 19.2% (173/901) of urban pregnant women attended their first antenatal check at later than 4 months of their gestational age.

Regarding the educational status of the study participants, 43.4% (1274/2935) of them had not ever attended school. Among those who ever attended school, 64.7% (1074/1661) attended primary school, 21.6% (359/1661) attended secondary school, 6.3% (105/1661) attended technical or vocational school and 7.4% (123/1661) attended higher education.

### Mean and mean difference in timing at first antenatal check across regions in Ethiopia

The mean timing of the first antenatal check in months among Ethiopian women was assessed. The mean (± SD) timing of the first antenatal check-ups among pregnant women in Ethiopia was 4.81 ± 9.11 months. The timing of the first antenatal check in Addis Ababa City Administration and the Harari region was relatively earlier than check-ups in other regions (Table 1).

**Table 1:** Mean and standard deviation in timing at first antenatal checks in months among women attending health facilities in Ethiopia, Mini DHS 2019

Socio-demographic characteristics	Timing of 1st antenatal check (months)			
Age in 5-year groups	Total Observation (N)	Mean ± Std. Deviation		
15–19	170	4.08	1.69	
20–24	614	4.91	10.12	
25–29	953	4.60	8.19	
30–34	603	5.02	10.20	
35–39	396	5.50	11.61	
40–44	154	4.17	1.53	
45–49	45	4.18	1.25	
**Type of place of residence**				
Urban	901	3.90	7.17	
Rural	2034	5.22	9.83	
**Region**				
Tigray	323	4.20	5.41	
Afar	231	4.90	8.89	
Amhara	337	6.26	14.42	
Oromia	346	4.80	7.28	
Somali	90	5.71	10.03	
Benishangul	300	5.22	10.89	
SNNPR	327	5.30	7.45	
Gambela	268	4.40	5.89	
Harari	250	3.97	8.56	
Addis Adaba	229	3.71	6.40	
Dire Dawa	234	4.43	10.79	
**Ever attended school**				
No	1274	5.68	11.48	
Yes	1661	4.15	6.69	
**Educational attainment**				
No education	1274	5.68	11.48	
Incomplete primary	915	4.47	7.10	
Complete primary	159	4.16	7.65	
Incomplete secondary	319	4.12	7.57	
Complete secondary	40	3.35	1.51	
Higher	228	3.04	1.19	
**Religion**				
Orthodox	1095	4.78	9.51	
Catholic	19	3.95	1.18	
Protestant	586	5.03	8.78	
Muslim	1212	4.76	9.07	
Traditional	15	4.60	1.45	
Other	8	4.50	1.19	
**Total**	**2935**	**4.81**	**9.11**	

Additionally, the results showed that timing at the first antenatal check in the Amhara region versus Addis Ababa was statistically significant at the p-value < 0.05 level, as indicated by *** in Table 2. The mean of timing at first antenatal check-ups in months was greater in the Amhara region than in the rest of the regions. Although there was a mean difference in the timing of the first antenatal check in all regions and two administrative cities, the mean difference was not statistically significant except for Amhara versus Addis Ababa and vice versa.

**Table 2:** Mean comparison among women attending the first antenatal check across regions in Ethiopia, mini DHS 2019

Region Comparison	Difference Between Means	95% Confidence interval		*P*-value
**Amhara - Somali**	0.5441	-2.9343	4.0224	1.000
**Amhara - SNNPR**	0.9555	-1.3201	3.2311	0.959
**Amhara - Benishangul**	1.0385	-1.2884	3.3655	0.939
**Amhara - Afar**	1.3504	-1.1537	3.8545	0.816
**Amhara - Oromia**	1.4517	-0.7919	3.6954	0.589
**Amhara - Dire Dawa**	1.8278	-0.6667	4.3224	0.393
**Amhara - Gambela**	1.8522	-0.5471	4.2516	0.313
**Amhara - Tigray**	2.0601	-0.2226	4.3429	0.121
**Amhara - Harari**	2.2832	-0.1638	4.7302	0.093
**Amhara - Addis Adaba**	2.5478	0.0372	5.0583	0.043***
**Somali - Amhara**	-0.5441	-4.0224	2.9343	1.000
**Somali - SNNPR**	0.4114	-3.0781	3.9010	1.000
**Somali - Benishangul**	0.4944	-3.0288	4.0177	1.000
**Somali - Afar**	0.8063	-2.8363	4.4490	1.000
**Somali - Oromia**	0.9076	-2.5612	4.3765	0.999
**Somali - Dire Dawa**	1.2838	-2.3524	4.9199	0.988
**Somali - Gambela**	1.3081	-2.2634	4.8796	0.985
**Somali - Tigray**	1.5161	-1.9782	5.0103	0.949
**Somali - Harari**	1.7391	-1.8646	5.3428	0.901
**Somali - Addis Adaba**	2.0037	-1.6435	5.6508	0.798
**SNNPR - Amhara**	-0.9555	-3.2311	1.3201	0.959
**SNNPR - Somali**	-0.4114	-3.9010	3.0781	1.000
**SNNPR - Benishangul**	0.0830	-2.2606	2.4267	1.000
**SNNPR - Afar**	0.3949	-2.1247	2.9145	1.000
**SNNPR - Oromia**	0.4962	-1.7647	2.7572	1.000
**SNNPR - Dire Dawa**	0.8723	-1.6378	3.3825	0.990
**SNNPR - Gambela**	0.8967	-1.5188	3.3123	1.000
**SNNPR - Tigray**	1.1046	-1.1951	3.4044	0.904
**SNNPR - Harari**	1.3277	-1.1352	3.7906	0.817
**SNNPR - Addis Adaba**	1.5923	-0.9338	4.1183	0.627
**Benishangul - Amhara**	-1.0385	-3.3655	1.2884	0.939
**Benishangul - Somali**	-0.4944	-4.0177	3.0288	1.000
**Benishangul - SNNPR**	-0.0830	-2.4267	2.2606	1.000
**Benishangul - Afar**	0.3119	-2.2542	2.8780	1.000
**Benishangul - Oromia**	0.4132	-1.8995	2.7259	1.000
**Benishangul - Dire Dawa**	0.7893	-1.7675	3.3461	0.996
**Benishangul - Gambela**	0.8137	-1.6503	3.2777	0.993
**Benishangul - Tigray**	1.0216	-1.3290	3.3722	0.949
**Benishangul - Harari**	1.2447	-1.2658	3.7551	0.884
**Benishangul - Addis Adaba**	1.5092	-1.0632	4.0817	0.724
**Afar - Amhara**	-1.3504	-3.8545	1.1537	0.816
**Afar - Somali**	-0.8063	-4.4490	2.8363	1.000
**Afar - SNNPR**	-0.3949	-2.9145	2.1247	1.000
**Afar - Benishangul**	-0.3119	-2.8780	2.2542	1.000
**Afar - Oromia**	0.1013	-2.3895	2.5921	1.000
**Afar - Dire Dawa**	0.4774	-2.2416	3.1964	1.000
**Afar - Gambela**	0.5018	-2.1302	3.1337	1.000
**Afar - Tigray**	0.7097	-1.8164	3.2358	0.988
**Afar - Harari**	0.9328	-1.7427	3.6082	0.989
**Afar - Addis Adaba**	1.1973	-1.5364	3.9310	0.946
**Oromia - Amhara**	-1.4517	-3.6954	0.7919	0.589
**Oromia - Somali**	-0.9076	-4.3765	2.5612	0.999
**Oromia - SNNPR**	-0.4962	-2.7572	1.7647	1.000
**Oromia - Benishangul**	-0.4132	-2.7259	1.8995	1.000
**Oromia - Afar**	-0.1013	-2.5921	2.3895	1.000
**Oromia - Dire Dawa**	0.3761	-2.1051	2.8573	1.000
**Oromia - Gambela**	0.4005	-1.9850	2.7860	0.985
**Oromia - Tigray**	0.6084	-1.6597	2.8766	0.999
**Oromia - Harari**	0.8315	-1.6019	3.2649	0.991
**Oromia - Addis Adaba**	1.0960	-1.4013	3.5934	0.945
**Dire Dawa - Amhara**	-1.8278	-4.3224	0.6667	0.393
**Dire Dawa - Somali**	-1.2838	-4.9199	2.3524	0.988
**Dire Dawa - SNNPR**	-0.8723	-3.3825	1.6378	0.990
**Dire Dawa - Benishangul**	-0.7893	-3.3461	1.7675	0.996
**Dire Dawa - Afar**	-0.4774	-3.1964	2.2416	1.000
**Dire Dawa - Oromia**	-0.3761	-2.8573	2.1051	1.000
**Dire Dawa - Gambela**	0.0244	-2.5985	2.6472	1.000
**Dire Dawa - Tigray**	0.2323	-2.2843	2.7489	1.000
**Dire Dawa - Harari**	0.4554	-2.2112	3.1219	1.000
**Dire Dawa - Addis Adaba**	0.7199	-2.0050	3.4449	0.999
**Gambela - Amhara**	-1.8522	-4.2516	0.5471	0.313
**Gambela - Somali**	-1.3081	-4.8796	2.2634	0.985
**Gambela - SNNPR**	-0.8967	-3.3123	1.5188	0.983
**Gambela - Benishangul**	-0.8137	-3.2777	1.6503	0.993
**Gambela - Afar**	-0.5018	-3.1337	2.1302	1.000
**Gambela - Oromia**	-0.4005	-2.7860	1.9850	1.000
**Gambela - Dire Dawa**	-0.0244	-2.6472	2.5985	1.000
**Gambela - Tigray**	0.2079	-2.2143	2.6302	1.000
**Gambela - Harari**	0.4310	-2.1467	3.0086	1.000
**Gambela - Addis Adaba**	0.6956	-1.9425	3.3337	0.999
**Tigray - Amhara**	-2.0601	-4.3429	0.2226	0.121
**Tigray - Somali**	-1.5161	-5.0103	1.9782	0.949
**Tigray - SNNPR**	-1.1046	-3.4044	1.1951	0.904
**Tigray - Benishangul**	-1.0216	-3.3722	1.3290	0.949
**Tigray - Afar**	-0.7097	-3.2358	1.8164	0.998
**Tigray - Oromia**	-0.6084	-2.8766	1.6597	0.999
**Tigray - Dire Dawa**	-0.2323	-2.7489	2.2843	1.000
**Tigray - Gambela**	-0.2079	-2.6302	2.2143	1.000
**Tigray - Harari**	0.2230	-2.2464	2.6925	1.000
**Tigray - Addis Adaba**	0.4876	-2.0449	3.0201	1.000
**Harari - Amhara**	-2.2832	-4.7302	0.1638	0.093
**Harari - Somali**	-1.7391	-5.3428	1.8646	0.991
**Harari - SNNPR**	-1.3277	-3.7906	1.1352	0.817
**Harari - Benishangul**	-1.2447	-3.7551	1.2658	0.884
**Harari - Afar**	-0.9328	-3.6082	1.7427	0.989
**Harari - Oromia**	-0.8315	-3.2649	1.6019	0.991
**Harari - Dire Dawa**	-0.4554	-3.1219	2.2112	1.000
**Harari - Gambela**	-0.4310	-3.0086	2.1467	1.000
**Harari - Tigray**	-0.2230	-2.6925	2.2464	1.000
**Harari - Addis Adaba**	0.2646	-2.4169	2.9461	1.000
**Addis Adaba - Amhara**	-2.5478	-5.0583	-0.0372	0.043***
**Addis Adaba - Somali**	-2.0037	-5.6508	1.6435	0.988
**Addis Adaba - SNNPR**	-1.5923	-4.1183	0.9338	0.990
**Addis Adaba - Benishangul**	-1.5092	-4.0817	1.0632	0.996
**Addis Adaba - Afar**	-1.1973	-3.9310	1.5364	0.946
**Addis Adaba - Oromia**	-1.0960	-3.5934	1.4013	0.945
**Addis Adaba - Dire Dawa**	-0.7199	-3.4449	2.0050	0.999
**Addis Adaba - Gambela**	-0.6956	-3.3337	1.9425	0.999
**Addis Adaba - Tigray**	-0.4876	-3.0201	2.0449	1.000
**Addis Adaba - Harari**	-0.2646	-2.9461	2.4169	1.000

Note: Comparisons significant at the 0.05 level are indicated by ***

### Correlation between different variables and timing at first antenatal check in Ethiopia

**Fig. 1:**
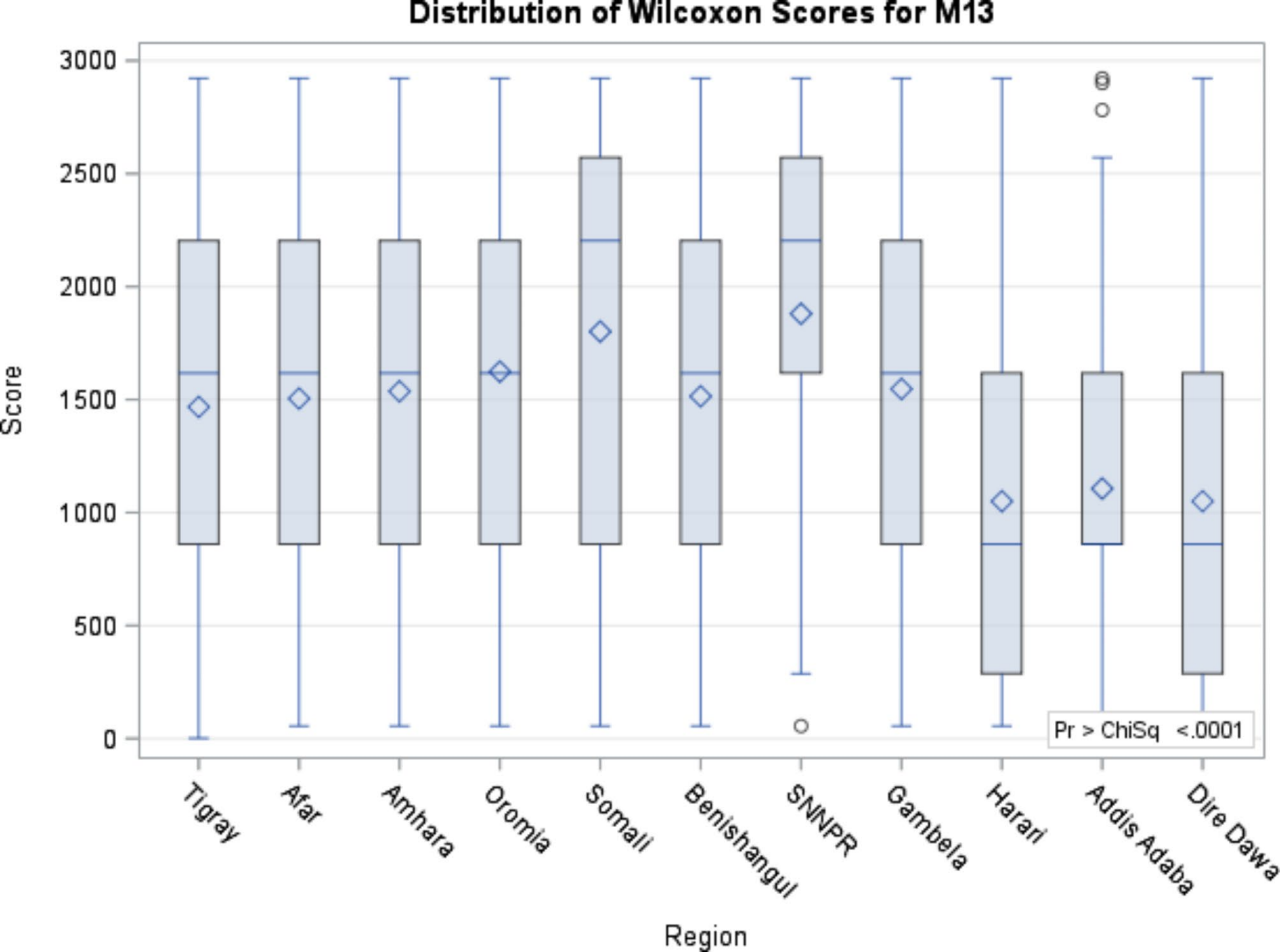
Distribution of Wilcoxon Kruskal- Wallis test for timing at the first antenatal check. Note: M13 = Timing at first antenatal checks

From table 3, we can understand that women’s previous place of delivery (r=-0.053, *p* = 0.004), previous antenatal care at government (r= -0.041, *p* = 0.027) and private facilities (r = -0.045, *p* = 0.014), ever attended school (r = -0.083, *p* = < 0.0001), and highest educational level (*r* = -0.069, *p* = 0.005) and counselling by health workers during a previous pregnancy (r = -0.098, *p* = < 0.0001) were negatively correlated with timing at first antenatal check-ups in months. All the correlations were significant at α < 0.05 (Table 3). Figure 1 shows the mean difference across regions of timing at the first antenatal check in Ethiopia (Fig. 1).

**Table 3:** Multiple correlation results among women attending antenatal check in Ethiopia, mini DHS 2019

Description	Timing at first antenatal checks		
	Observation	Correlation	*P*-value
Place of delivery	2935	-0.053	0.004
Delivery by caesarean section	2935	-0.027	0.140
Antenatal care: government hospital	2935	-0.041	0.027
Antenatal care: private hospital	2935	-0.045	0.014
Ever attended school	2935	-0.083	< 0.0001
Highest educational level	1661	-0.069	0.005
Religion	2935	-0.002	0.934
Region	2935	-0.033	0.075
During pregnancy: counselling by health worker	2935	-0.098	< 0.0001

### ANOVA and multiple linear regression results

Multiple Linear regression was conducted to test whether previous delivery, previous antenatal care at both government and private facilities, ever-attended school, and highest educational level and counselling by health workers during previous pregnancy significantly predicted the timing of the first antenatal check of women in nine different regions and two city administrations (Table 4). The results of the analysis suggest that the regression model significantly predicts timing at the first antenatal check (*p* = 0.001). Counselling by health workers during previous pregnancy significantly predicts the timing at first antenatal checks in months holding previous delivery, previous antenatal care at both government and private facilities, ever attended school, and highest educational level. Timing at the first antenatal check in months is expected to decrease by 0.99 months for every counselling session at each pregnancy in a previous antenatal check.

**Table 4:** Multiple linear regression result for timing at first antenatal check in Ethiopia, mini DHS 2019

Variables	Unadjusted Regression Coefficient	Adjusted Regression Coefficient	Standard Error	*P*-value
During pregnancy: counselling health worker	-1.97055	-0.99058	0.38346	0.0099
Place of delivery	-0.05053	-0.01852	0.01987	0.3514
Delivery by caesarean section	-0.85621	-0.83247	0.52155	0.1106
Antenatal care: government hospital	-1.12308	-0.48052	0.46442	0.3010
Antenatal care: private hospital	-2.13333	-1.10595	0.70911	0.1190
Highest grade/form/year at that level	-0.01363	0.00666	0.05487	0.9035
Religion	-0.00275	0.01195	0.02652	0.6524
Type of place of residence	1.31337	-0.01541	0.36693	0.9665

## Discussion

The mean difference in the timing of the first antenatal check of women among 10 regions in Ethiopia was analysed using ANOVA, and it was found that the mean timing of the first antenatal check in months was greatest in Amhara, Somalia, SNNPR, Benishangul, Afar, Oromia, Dire Dawa, Gambela, Tigray, Harari, and Addis Ababa. The first antenatal check for women was timed earlier in Addis Ababa than in the rest of the region. Although the mean difference in timing at first antenatal checks in all regions except Addis Ababa appeared delayed, it became higher in the Amhara region. The mean difference between Addis Ababa and Amhara regarding the first ANC visits was found statistically significant. This is consistent with the findings of utilisation and findings of antenatal care visits in East African countries(14), where domestic child-rearing, agricultural activities, access and demand for additional costs were reported as causes of delays in timely ANC visits. Furthermore, the disagreement in the first ANC visits and the discrepancy across each region of the study area are concurrent with existing global and national facts that are determined by whether residents are urban or rural. A previous report from an Ethiopian demographic health survey demonstrated that, only 20% of pregnant mothers started their antenatal care visits in the first trimester of pregnancy, and most (44%) of those mothers lived in urban areas, while only 17% of those who started ANC visits in the first trimester of pregnancy lived in rural communities [21]. Accordingly, the current result is in agreement with such a report, as particularly Addis Ababa is the largest city in Ethiopia and the capital where better education, awareness and professional counselling could be provided to receive their first antenatal service earlier than in other regions of the country. Several factors have been found to hinder timely ANC visits. It was found that maternal age, maternal education, husband’s education, maternal occupation, place of residence, parity, knowledge of ANC, women’s autonomy, partner involvement, pregnancy intention, presence of pregnancy complications, means of identifying pregnancy, employment, incomes, differences in the mother’s age, and the degree of women’s autonomy significantly contributed to the delayed initiation of ANC in Ethiopia.

These results are consistent with results of antenatal care booking within the first trimester of pregnancy and its associated factors among pregnant women residing in an urban area in Debre Berhan town, timing of first antenatal care attendance and associated factors among pregnant women in public health institutions of Axum town, Tigray, delayed initiation of antenatal care and associated factors in Ethiopia, time to initiation of antenatal care and its predictors among pregnant women in Ethiopia, timing of first antenatal care visit and its associated factors among pregnant women attending public health facilities in Addis Ababa and timing of first antenatal care visits and number of items of antenatal care contents received and associated factors in Ethiopia on factors affecting timely ANC attendance [10–12, 22, 23]. The timing of the first antenatal check was negatively correlated with the place of previous delivery, previous antenatal care at both government and private facilities, having ever attended school at the highest educational level and counselling provided by a health worker during the previous pregnancy. This suggests that there is a need to improve access and awareness of antenatal care services among women who have had previous deliveries, limited education, and little exposure to health education, as confirmed by the work of Pervin [15] and Tripathy [16], in which facility deliveries were associated with woman’s age, parity, education, husband’s education, and wealth index. The timing of the first antenatal check is significantly impacted by counselling provided by a health professional during a prior pregnancy. When other variables were adjusted, however, timing at the first antenatal check was not significantly associated with previous delivery location, previous caesarean delivery, prior antenatal care received at government and private health facilities, educational level, religious affiliation, or type of residence. This finding was in line with a study conducted by Alemu et al., implying that prenatal counselling provided by healthcare professionals can be extremely helpful in encouraging women to seek antenatal care earlier in subsequent pregnancies [16, 18, 19, 24]. However, other studies reported that income, residence, women’s occupational status, transportation payment, and access to information about the first timing of ANC are associated with the first timing of ANC [9, 25]. Therefore, several variables that were not included in the current analysis such as unintended pregnancy and others, may be responsible for the delayed first-time ANC in most of the Ethiopian regional states in general and the Amhara region in particular. For instance, women living in urban areas were 80% less likely to delay to first ANC visits than those living in rural areas, and husbands’ occupation such as being a farmer or government employee can also be another predictor variable for delays in the first checks [9, 23]. The delayed time of first antenatal care attendance in Ethiopia, as found in this research, agrees with previous findings, where delayed timely ANC from sub-Saharan Africa was reported [13, 19, 26–28]. The World Health Organization recently recommends eight antenatal visits for women in its new guidelines comprising interventions such as tetanus toxoid vaccination, screening and treatment for infections, and identification of warning signs during pregnancy [4]. National guidelines for ANC in Ethiopia also recommend that every pregnant woman is expected to receive ANC from a skilled health service provider. This includes a comprehensive physical examination, blood tests for screening of anaemia and infection, a urine test, tetanus toxoid injections, iron and foliate supplements, and medications for deworming [5]. Moreover, the WHO has designed a new ANC model that advises, pregnant women to make their first contact with healthcare professionals within the first 12 weeks of gestation. This research has revealed Ethiopia’s low compliance with ANC utilization at the national level, and different milestone measures to cope with the WHO recommendation are highly important, which is in line with the low compliance with ANC utilization in both, national and WHO guidelines, as confirmed by earlier reports from different parts of Ethiopia [23, 25, 29].

## Conclusion

The mean difference in timing at the first antenatal check in months among Ethiopian pregnant women in the two regions significantly varied. Women’s previous place of delivery, previous antenatal care at government and private facilities, ever attended school, and highest educational level and counselling by health workers during previous pregnancy were negatively correlated with timing at first antenatal check-ups in months. Previous pregnancy counselling by health workers positively influenced the timing of first antenatal check-ups for subsequent antenatal follow-ups among pregnant women in Ethiopia. Moreover, the first antenatal check for women was earlier in Addis Ababa than in all other regional states of the country. Working on the timing at the first antenatal check in the earliest possible in the rest of the regions in Ethiopia was recommended as a strategy for the early antenatal check.

## Data Availability

The data that support the findings of this study are available from the Demographic Health Survey (DHS) 2019, but restrictions apply to the availability of these data, which were used under license for the current study and so are not publicly available. Data are, however, available from the corresponding author upon reasonable request and with the permission of DHS.

## References

[1] Villar J, Ba’aqeel H, Piaggio G, Lumbiganon P, Belizán JM, Farnot U (2001). WHO antenatal care randomised trial for the evaluation of a new model of routine antenatal care. Lancet.

[2] United Nations Maternal Mortality Estimation Inter-agency Group. (2023). World Health Organization. Trends in maternal mortality 2000 to 2020. 2020.

[3] Bianchi-Jassir F, Seale AC, Kohli-Lynch M, Lawn JE, Baker CJ, Bartlett L et al. Preterm Birth Associated With Group B Streptococcus Maternal Colonization Worldwide: Systematic Review and Meta-analyses. Clin Infect Dis. 2017/11/09. 2017;65(suppl_2):S133-s142. 10.1093/cid/cix661 PMC5850429 29117329

[4] World Health Organization. 2016. WHO recommendations on antenatal care for a positive pregnancy experience. 28079998

[5] Ministry of Health-Ethiopia. National Antenatal Care. 2022.

[6] Ornella L, Seipati M-AP. Opportunities for Africa ’ s Newborns.

[7] Makate M, Makate C (2017). Prenatal care utilization in Zimbabwe: examining the role of community-level factors. J Epidemiol Glob Health.

[8] Fentaw KD, Fenta SM, Biresaw HB (2021). Time to first antenatal care visit among pregnant women in Ethiopia: secondary analysis of EDHS 2016; application of AFT shared frailty models. Arch Public Heal.

[9] Asnake G, Lalu H, Birhanu T, Guye B. Pregnant women attending Antenatal Care and First Antenatal Care visit in Ethiopia. 2021;18:498–505.

[10] Tesfaye G, Loxton D, Chojenta C, Semahegn A, Smith R. Delayed initiation of antenatal care and associated factors in Ethiopia: a systematic review and meta-analysis. 2017; 14 (1):150. 10.1186/s12978-017-0412-4. 10.1186/s12978-017-0412-4 PMC5688656 29141675

[11] Woldeamanuel BT, Belachew TA (2021). Timing of first antenatal care visits and number of items of antenatal care contents received and associated factors in Ethiopia: multilevel mixed effects analysis. Reprod Health.

[12] Hanna G, Gulema H, Berhane Y. Timing of First Antenatal Care Visit and its Associated factors among Pregnant Women Attending Public Health Facilities in Addis. 10.4314/ejhs.v27i2.6. 10.4314/ejhs.v27i2.6 PMC5440828 28579709

[13] Okedo-alex IN, Akamike IC, Ezeanosike OB et al. Determinants of antenatal care utilisation in sub-saharan Africa: a systematic review. 2019; 10.1136/bmjopen-2019-031890. 10.1136/bmjopen-2019-031890 PMC6797296 31594900

[14] Alene AG, Olayemi OO, Berhane Y (2021). Timing and factors associated with early antenatal visits among pregnant women in west Gojjam, northwest Ethiopia. Afr J Midwifery Womens Health.

[15] Pervin J, Venkateswaran M, Nu UT, Rahman M, O’Donnell BF, Friberg IK et al. Determinants of utilization of antenatal and delivery care at the community level in rural Bangladesh. PLoS One. 2021;16(9):e0257782. 10.1371/journal.pone.0257782. eCollection 2021. 10.1371/journal.pone.0257782 PMC8478219 34582490

[16] Tripathy A, Mishra PS (2023). Inequality in time to first antenatal care visits and its predictors among pregnant women in India: an evidence from national family health survey. Sci Rep.

[17] Kassebaum N, Kyu HH, Zoeckler L, Olsen HE, Thomas K, Pinho C et al. Child and Adolescent Health From 1990 to 2015: Findings From the Global Burden of Diseases, Injuries, and Risk Factors 2015 Study. JAMA Pediatr. 2017/04/07. 2017;171(6):573–92. 10.1001/jamapediatrics.2017.0250. 10.1001/jamapediatrics.2017.0250 PMC5540012 28384795

[18] Alemu Y, Aragaw A. Early initiations of first antenatal care visit and associated factor among mothers who gave birth in the last six months preceding birth in Bahir Dar Zuria Woreda North West Ethiopia. Reprod Health. 2018; Dec 12;15(1):203. 10.1186/s12978-018-0646-9. 10.1186/s12978-018-0646-9 PMC6292069 30541562

[19] Yakubu I, Salisu WJ. Determinants of adolescent pregnancy in sub-Saharan Africa: a systematic review. Reprod Health. 2018; Dec 27;15(1):15. Dio: 10.1186/s12978-018-0460-4. 10.1186/s12978-018-0460-4 PMC5787272 29374479

[20] Ethiopian Public Health Institute (EPHI) [Ethiopia] and ICF. 2021. Ethiopia Mini Demographic and Health Survey 2019: Final Report. Rockville, Maryland, USA: EPHI and ICF. 2019.

[21] Ethiopian Central Statistics Agency. (ECSA 2016). Ethiopia Demographic and Health Survey 2016. Addis Ababa, Ethiopia, and Rockville, Maryland, USA: CSA and ICF. 2016.

[22] Kolola T, Morka W, Abdissa B. Antenatal care booking within the first trimester of pregnancy and its associated factors among pregnant women residing sectional study in an urban area: a cross- ­ in Debre Berhan town, Ethiopia. 2020;1–6. 10.1136/bmjopen-2019-032960. 10.1136/bmjopen-2019-032960 PMC7311019 32571853

[23] Gebresilassie B, Belete T, Tilahun W et al. Timing of first antenatal care attendance and associated factors among pregnant women in public health institutions of Axum town, Tigray, Ethiopia, 2017 : a mixed design study. 2019;5:1–11. 10.1186/s12884-019-2490-5. 10.1186/s12884-019-2490-5 PMC6751589 31533657

[24] Paudel YR, Jha T, Mehata S. Timing of First Antenatal Care (ANC) and inequalities in early initiation of ANC in Nepal. Front Public Heal. 2017;5. 10.3389/fpubh.2017.00242. 10.3389/fpubh.2017.00242 PMC5600995 28955707

[25] Dewau R, Muche A, Fentaw Z, Yalew M, Bitew G, Amsalu ET (2021). Time to initiation of antenatal care and its predictors among pregnant women in Ethiopia: Cox-gamma shared frailty model. PLoS ONE.

[26] Gudissa Damme T (2015). Time of Antenatal Care Booking and Associated Factors among Pregnant Women Attending Ambo Town Health Facilities, Central Ethiopia. J Gynecol Obstet.

[27] Ritbano Ahmed M, Sultan S, Abose et al. Levels and associated factors of the maternal healthcare continuum in Hadiya zone, Southern Ethiopia : A multilevel analysis. 2022;1–18. 10.1371/journal.pone.0275752. 10.1371/journal.pone.0275752 PMC9550044 36215257

[28] Aboagye RG, Okyere J, Ahinkorah BO, Seidu A-A, Zegeye B, Amu H (2022). Health insurance coverage and timely antenatal care attendance in sub-saharan Africa. BMC Health Serv Res.

[29] Kasiye Shiferaw B, Mengiste, Tesfaye Gobena MD. The effect of antenatal care on perinatal outcomes in Ethiopia: A systematic review. 2021;42:1–19. 10.1371/journal.pone.0245003. 10.1371/journal.pone.0245003 PMC7808692 33444374

